# Multi-symplectic integrator of the generalized KdV-type equation based on the variational principle

**DOI:** 10.1038/s41598-019-52419-8

**Published:** 2019-11-04

**Authors:** Yi Wei, Xing-Qiu Zhang, Zhu-Yan Shao, Jian-Qiang Gao, Xiao-Feng Yang

**Affiliations:** 1 School of Medical Information Engineering, Jining Medical University, Rizhao, 276826 Shandong P.R. China; 2 College of Science, Northwest A&F University, Yangling, 712100 Shaanxi P. R. China

**Keywords:** Mathematics and computing, Physics

## Abstract

The variational principle is used to construct a multi-symplectic structure of the generalized KdV-type equation. Accordingly, the local energy conservation law, the local momentum conservation law, and the Cartan form of the generalized KdV-type equation are given. An explicit multi-symplectic scheme for the generalized KdV equation based on the Fourier pseudo-spectral method and the symplectic Euler scheme is constructed. Through a numerical examination, the explicit multi-symplectic Fourier pseudo-spectral scheme for the generalized KdV equation not only preserve the discrete global energy conservation law and the global momentum conservation law with high accuracy, but show long-time numerical stability as well.

## Introduction

In this paper, we aim to study the generalized KdV-type equation in the form

1.1 $${u}_{t}+f(u){u}_{x}+{(g({u}_{x}))}_{xx}=0,$$

where *f* and *g* are smooth functions.

When *f*(*u*) = *αu*^*λ*^ and *g*(*u*_*x*_) = *δu*_*x*_, Eq. (1.1) reduces to the generalized KdV equation

1.2 $${u}_{t}+\alpha {u}^{\lambda }{u}_{x}+\delta {u}_{xxx}=0,$$

where *α*,*δ*, and *λ* are arbitrary constants.

Setting *λ* = 1, Eq. (1.2) reduces to the KdV equation^1,2^

1.3 $${u}_{t}+\alpha u{u}_{x}+\delta {u}_{xxx}=0.$$

Setting *λ* = 2, Eq. (1.2) reduces to the mKdV equation^1,2^

1.4 $${u}_{t}+\alpha {u}^{2}{u}_{x}+\delta {u}_{xxx}=0.$$

The KdV equation is originally used to describe long waves propagating in a channel. The KdV equation can also describes the propagation of plasma waves in a dispersive medium^2,3^. The KdV equation and the mKdV equation are most popular soliton equations which have been extensively studied^2,4–11^, because these two equations possess many interesting properties of mechanism and geometry^12^. Based on the homogeneous balance of undetermined coefficients method (HBUCM)^13,14^, Yang *et al*.^13^ proposed the definition and a multi-symplectic structure of the generalized KdV-type equation. Obviously, the KdV equation, the mKdV equation and the generalized KdV equation are special cases of the generalized KdV-type equation.

To understand the mechanism of complex physical phenomena, we should obtain solutions of the generalized KdV-type equation. There are some methods to obtain exact solutions, such as the first integral method^15^, the (*G*′/*G*)-expansion method^9^, the homogeneous balance method^16^, the modified simple equation method^17^, simplified Hirota’s method^18^, and so on. However, the generalized KdV-type equation is a nonlinear partial differential equation (NLPDE), it is difficult to obtain the exact solutions or there is no exact solution. In these cases, it is natural to resort to the numerical methods^13^. A large amount of numerical methods have been applied on the KdV equation and the mKdV equation in the last few years, including quintic B-Spline basis functions^19^, finite difference scheme^20^, symplectic method^12^, multi-symplectic Preissmann box scheme^6^, multi-symplectic box schemes^21^, and so on.

Many physical properties of a system are closely related to the geometric structure of the equation^22^. This naturally requires that numerical methods can preserve exactly geometric structure during the simulation. Multi-symplectic method possesses the stability and effectiveness, and can preserve the multi-symplectic structure of the Hamiltonian system^14,23^. In the present paper, a multi-symplectic structure of the generalized KdV-type equation is given by the variational principle. To obtain a multi-symplectic structure of the generalized KdV-type equation based on variational principle, we need to cast Eq. (1.1) into a system of equations. Therefore, it will be helpful to give a detailed derivation of a multi-symplectic structure for the generalized KdV-type equation since this derivation will provide some guiding principles in finding multi-symplectic structures for other NLPDEs.

We also consider a multi-symplectic Fourier pseudo-spectral discretization for Eq. (1.2) and demonstrate its convergence by simulating the evolution of the soliton. The remainder of this paper is organized as follows. In section 2, a multi-symplectic structure of the generalized KdV-type equation is derived based on the variational principle. An explicit multi-symplectic Fourier pseudo-spectral scheme for the generalized KdV equation is given in section 3. In section 4, the solitary wave behaviors of the generalized KdV equation are simulated. In section 5, some conclusions are given.

## Multi-Symplectic Structure of The Generalized Kdv-Type Equation Based on The Variational Principle

In this section, a multi-symplectic structure of the generalized KdV-type equation is given by the variational principle. The covariant configuration space for Eq. (1.1) is denoted by *X* × *U*, where *X* = (*x*, *t*) represents the space of independent variables and *U* = (*ϕ*, *u*, *v*, *ω*, *σ*) represents the space of dependent variables. The internal variables *ϕ*, *u*, *v*, *ω*, and *σ* are defined to construct a multi-symplectic structure of the generalized KdV-type equation. The first order prolongation of *X* × *U* is defined to be *U*^(1)^ = *X* × *U* × *U*_1_, where *U*_1_ = (*ϕ*, *u*, *v*, *ω*, *σ*, *ϕ*_*x*_, *u*_*x*_, *v*_*x*_, *ω*_*x*_, *σ*_*x*_, *ϕ*_*t*_, *u*_*t*_, *v*_*t*_, *ω*_*t*_, *σ*_*t*_) represents the space consisting of the first order partial derivatives. Let *ς*: *X* → *U* be a smooth function and we suppose *ς* ∈ *H*^2^[*X*] × *H*^2^[*X*], where *H*^2^[*X*] is the second order Sobolev space defined on *X*. Then, its first prolongation is denoted by ${\mathrm{pr}}^{1}\varsigma =(\varphi ,u,v,\omega ,\sigma ,{\varphi }_{x},{u}_{x},{v}_{x},{\omega }_{x},{\sigma }_{x},{\varphi }_{t},{u}_{t},{v}_{t},{\omega }_{t},{\sigma }_{t})$. The Lagrangian density for Eq. (1.1) is

2.1 $${L}({{\rm{pr}}}^{1}(\varsigma ))=L({{\rm{pr}}}^{1}(\varsigma ))dx\wedge dt,$$

where

2.2 $$L({{\rm{pr}}}^{1}(\varsigma ))=-\,2\iint f(u){d}^{2}u-u\omega -u{\varphi }_{t}+2\sigma {u}_{x}+{\varphi }_{x}\omega +2\int g(v)dv-2\sigma v.$$

Corresponding to the Lagrangian density (2.2), the action functional is defined by

2.3 $$S(\varsigma )={\int }_{{M}}{L}({{\rm{pr}}}^{1}(\varsigma )),$$

where *ς* ∈ *H*^2^[*M*] × *H*^2^[*M*] and *M* is an open set in *X*.

Let *V* be a vector field on *X* × *U* with the form

2.4 $$\begin{array}{c}V=\xi (x,t)\frac{\partial }{\partial t}+\eta (x,t)\frac{\partial }{\partial x}+\tau (x,t,\varphi ,u,v,\omega ,\sigma )\frac{\partial }{\partial \varphi }+\phi (x,t,\varphi ,u,v,\omega ,\sigma )\frac{\partial }{\partial u}\\ \,\,+\vartheta (x,t,\varphi ,u,v,\omega ,\sigma )\frac{\partial }{\partial v}+\rho (x,t,\varphi ,u,v,\omega ,\sigma )\frac{\partial }{\partial \omega }+\gamma (x,t,\varphi ,u,v,\omega ,\sigma )\frac{\partial }{\partial \sigma }.\end{array}$$

The flow exp(*βV*) of the vector field *V* is a one-parameter transformation group of *X* × *U*. The map *ς*: *M* → *U* and a family of maps $\tilde{\varsigma }:\tilde{{M}}\to U$ depend on the parameter *β*. Now, the variation of the action functional (2.1) is calculated as follows:

2.5 $$\begin{array}{rcl}\delta S & = & {\frac{d}{d\beta }|}_{\beta =0}S(\tilde{\varsigma })\\  & = & {\frac{d}{d\beta }|}_{\beta =0}{\int }_{\tilde{{M}}}{L}({{\rm{pr}}}^{1}(\tilde{\varsigma }))\\  & = & {\frac{d}{d\beta }|}_{\beta =0}{\int }_{\tilde{{M}}}(-\,2\iint f(\tilde{u}){d}^{2}\tilde{u}-\tilde{u}\tilde{\omega }-\tilde{u}{\tilde{\varphi }}_{\tilde{t}}+2\tilde{\sigma }{\tilde{u}}_{\tilde{x}}\\  &  & +\,{\tilde{\varphi }}_{\tilde{x}}\tilde{\omega }+2\int g(\tilde{v})d\tilde{v}-2\tilde{\sigma }\tilde{v})d\tilde{x}\wedge d\tilde{t}\\  & = & {\int }_{{M}}Adx\wedge dt+B,\end{array}$$

where

2.6 $$\begin{array}{c}A=\xi ({E}_{t}+{F}_{x})+\eta ({I}_{t}+{G}_{x})+\tau ({u}_{t}-{\omega }_{x})+\phi (-{\varphi }_{t}-2{\sigma }_{x}-2\int f(u)du-\omega )\\ \,\,\,+\,\vartheta (2g(v)-2\sigma )+\rho ({\varphi }_{x}-u)+\gamma (2{u}_{x}-2v),\end{array}$$

2.7 $$B={\int }_{\partial {M}}(\xi (Edx-Fdt)+\eta (Idx-Gdt)+\tau (\omega dt+udx)+\phi (2\sigma dt)),$$

2.8 $$\begin{array}{c}E=2\iint f(u){d}^{2}u-2\int g(v)dv,F=\omega {\varphi }_{t}+2\sigma {u}_{t},I=-\,{u}^{2},\\ G=2\iint f(u){d}^{2}u+u\omega +u{\varphi }_{t}-2\int g(v)dv+2\sigma v.\end{array}$$

If *ξ*, *η*, *τ*, *ϕ*, *ϑ*, *ρ*, and *γ* have compact support on *M*, then *B* = 0. In this case, with the requirement of *δS* = 0 and from Eq. (2.5), the variation *ξ* yields the local energy conservation law

2.9 $${E}_{t}+{F}_{x}=0,$$

and the variation *η* yields the local momentum conservation law

2.10 $${I}_{t}+{G}_{x}=0,$$

where *E*, *F*, *I*, and *G* are same as to Eq. (2.8).

For a conservative *L*, i.e., one that does not depend on *x* and *t* explicitly, Eqs (2.9) and (2.10) become the local energy conservation law and the local momentum conservation law, respectively^5^.

The variations *τ*, *ϕ*, *ϑ*, *ρ*, and *γ* yield the Euler-Lagrange equation

2.11 $$\begin{array}{c}\,{u}_{t}-{\omega }_{x}=0,\\ -{\varphi }_{t}-2{\sigma }_{x}=2\int f(u)du+\omega ,\\ \,\,\,0=2\sigma -2g(v),\\ \,\,\,{\varphi }_{x}=u,\\ \,\,\,2{u}_{x}=2v.\end{array}$$

If the condition that *ξ*, *η*, *τ*, *σ*, *θ*, *ϑ*, and *ς* having compact support on *M* is not imposed, then from the boundary integral *B*, the Cartan form can be defined as

2.12 $$\begin{array}{c}{\varTheta }_{{L}}=(\omega d\varphi +2\sigma du)\wedge dt+ud\varphi \wedge dx\\ \,\,+(2\int g(v)dv-2\iint f(u){d}^{2}u-u\omega -2\sigma v)dx\wedge dt,\end{array}$$

which satisfies (denote the interior product and pull back mapping as ⌋ and ()*)

2.13 $$B={\int }_{\partial {M}}{({{\rm{pr}}}^{1}\varsigma )}^{\ast }\langle {{\rm{pr}}}^{1}V\_\,|{\varTheta }_{{L}}\rangle .$$

The multi-symplectic form of the generalized KdV-type equation is defined to be

2.14 $${\varTheta }_{{L}}=d{\varTheta }_{{L}}.$$

### Remark 1

Equation (2.11) is equivalent to a multi-symplectic structure of generalized KdV-type Eq. (1.1) as follows:

2.15 $${{\boldsymbol{M}}}_{5}{z}_{t}+{{\boldsymbol{K}}}_{5}{{\boldsymbol{z}}}_{x}={\nabla }_{{\boldsymbol{z}}}S({\boldsymbol{z}}),$$

where

$${{\boldsymbol{M}}}_{5}=[\begin{array}{ccccc}0 & 1 & 0 & 0 & 0\\ -\,1 & 0 & 0 & 0 & 0\\ 0 & 0 & 0 & 0 & 0\\ 0 & 0 & 0 & 0 & 0\\ 0 & 0 & 0 & 0 & 0\end{array}],{{\boldsymbol{K}}}_{5}=[\begin{array}{ccccc}0 & 0 & 0 & -\,1 & 0\\ 0 & 0 & 0 & 0 & -2\\ 0 & 0 & 0 & 0 & 0\\ 1 & 0 & 0 & 0 & 0\\ 0 & 2 & 0 & 0 & 0\end{array}],{\boldsymbol{z}}=[\begin{array}{c}\varphi \\ u\\ v\\ \omega \\ \sigma \end{array}],$$

and Hamiltonian function $S({\boldsymbol{z}})=u\omega +2\sigma v-2\int g(v)dv+2\iint f(u){d}^{2}u.$

### **Remark 2**.

The multi-symplectic structure (2.15) is identical to the results by using the HBUCM^13^. Moreover, the Cartan form of the generalized KdV-type equation can be obtained by the variational principle.

### **Remark 3**.

Obviously, a multi-symplectic structure of the generalized KdV Eq. (1.2) is

2.16 $$\begin{array}{c}\,\,{u}_{t}-{\omega }_{x}=0,\\ -{\varphi }_{t}-2\delta {v}_{x}=\frac{2\alpha {u}^{\lambda +1}}{(\lambda +1)}+\omega ,\\ \,\,2\delta {u}_{x}=2\delta v0\\ \,\,\,{\varphi }_{x}=u,\end{array}$$

or

2.17 $${{\boldsymbol{M}}}_{4}{{\boldsymbol{z}}}_{t}+{{\boldsymbol{K}}}_{4}{{\boldsymbol{z}}}_{x}={\nabla }_{{\boldsymbol{z}}}S({\boldsymbol{z}}),$$

where

$${{\boldsymbol{M}}}_{4}=[\begin{array}{cccc}0 & 1 & 0 & 0\\ -\,1 & 0 & 0 & 0\\ 0 & 0 & 0 & 0\\ 0 & 0 & 0 & 0\end{array}],{{\boldsymbol{K}}}_{4}=[\begin{array}{cccc}0 & 0 & 0 & -\,1\\ 0 & 0 & -\,2\delta  & 0\\ 0 & 2\delta  & 0 & 0\\ 1 & 0 & 0 & 0\end{array}],{\boldsymbol{z}}=[\begin{array}{c}\varphi \\ u\\ v\\ \omega \end{array}],$$

and Hamiltonian function $S({\boldsymbol{z}})=\frac{2\alpha {u}^{\lambda +2}}{({\lambda }^{2}+3\lambda +2)}+\delta {v}^{2}+u\omega$.

According to the multi-symplectic theory presented by Bridges^24–27^, the multi-symplectic conservation law in the wedge product form, the local energy conservation law and the local momentum conservation law for the generalized KdV Eq. (1.2) are as follows:

2.18 $${\theta }_{t}+{\kappa }_{x}=0,{E}_{t}+{F}_{x}=0,{G}_{x}+{I}_{t}=0,$$

where

$$\theta =d\varphi \wedge du,\kappa =-\,d\varphi \wedge d\omega -2\delta du\wedge dv,$$

$$E=\frac{2\alpha {u}^{\lambda +1}}{{\lambda }^{2}+3\lambda +2}-\delta {v}^{2},F={\varphi }_{t}\omega +2\delta v{u}_{t},G=\frac{2\alpha {u}^{\lambda +2}}{({\lambda }^{2}+3\lambda +2)}+\delta {v}^{2}+u\omega +u{\varphi }_{t},I=-\,{u}^{2}.$$

## Explicit Multi-Symplectic Fourier Pseudo-Spectral Method For The Generalized Kdv Equation

In order to derive the algorithms conveniently, we introduce some notations: *x*_*i*_ = *x*_*L*_ + *ih*, *t*_*j*_ = *jτ* (*i* = 0, 1,…, *N* − 1; *j* = 0,1, …), where $h=\frac{{x}_{R}-{x}_{L}}{N}=\frac{L}{N}$ and *τ* are spatial and temporal step lengths, the indexes *i* and *j* denote the discrete space and time dimensions. Denote *u*_*i*_^*j*^ as the approximate value of *u*(*x*_*i*_, *t*_*j*_). As we know, the first-order differential operator ∂_*x*_ yields the Fourier spectral differentiation matrix ***D***. Here, ***D*** is an *N*×*N* anti-symmetry matrix with elements (*N* is an even number)

3.1 $${({\boldsymbol{D}})}_{r,s}=\{\begin{array}{c}\frac{1}{2}\mu {(-1)}^{r+s}\,\cot (\mu \frac{{x}_{r}-{x}_{s}}{2}),r\ne s,\\ 0,\,\,\,\,\,\,\,\,\,\,r=s,\end{array}$$

where *r* = 1,2, …, *N* and *s* = 1, 2,…, *N* represent column and row of the matrix ***D***, and $\mu =\frac{2\pi }{L}$. For more details, one can consult refs. ^11,28^ and references therein.

Using the notations

32 $${\boldsymbol{\Phi }}=[\begin{array}{c}{\varphi }_{0}\\ {\varphi }_{1}\\ \vdots \\ {\varphi }_{N-1}\end{array}],{\boldsymbol{u}}=[\begin{array}{c}{u}_{0}\\ {u}_{1}\\ \vdots \\ {u}_{N-1}\end{array}],{\boldsymbol{v}}=[\begin{array}{c}{v}_{0}\\ {v}_{1}\\ \vdots \\ {v}_{N-1}\end{array}],{\boldsymbol{\omega }}=[\begin{array}{c}{\omega }_{0}\\ {\omega }_{1}\\ \vdots \\ {\omega }_{N-1}\end{array}],{{\boldsymbol{u}}}^{\lambda +1}=[\begin{array}{c}{({u}_{0})}^{\lambda }\\ {({u}_{1})}^{\lambda }\\ \vdots \\ {({u}_{N-1})}^{\lambda }\end{array}],$$

and discretizing the multi-symplectic structure of the generalized KdV Eq. (2.17) with the Fourier pseudo-spectral method in the space domain, the discrete form of the generalized KdV Eq. (1.2) can be obtained as follows:

33 $$\begin{array}{c}\,\,\frac{d{\boldsymbol{u}}}{dt}-D{\boldsymbol{\omega }}=0,\\ -\,\frac{d{\boldsymbol{\Phi }}}{dt}-2\delta {\boldsymbol{Dv}}=\frac{2\alpha {{\boldsymbol{u}}}^{\lambda +1}}{\lambda +1}+{\boldsymbol{\omega }},\\ \,\,\,2\delta {\boldsymbol{Du}}=2\delta {\boldsymbol{v}},\\ \,\,\,{\boldsymbol{D}}{\boldsymbol{\Phi }}={\boldsymbol{u}}.\end{array}$$

### **Theorem 1**.

The Fourier pseudo-spectral semi-discretization (3.3) has *N* semi-discrete multi-symplectic conservation laws

3.4 $$\frac{d{{\boldsymbol{\chi }}}_{i}}{dt}+\mathop{\sum }\limits_{k=0}^{N-1}{({\boldsymbol{D}})}_{i,k}{{\boldsymbol{\zeta }}}_{i,k}=0,(i=1,2,\cdots ,N),$$

where ${{\boldsymbol{\chi }}}_{i}=\frac{1}{2}d{{\boldsymbol{z}}}_{i}\wedge {\boldsymbol{M}}d{{\boldsymbol{z}}}_{i},{{\boldsymbol{\zeta }}}_{i}=\frac{1}{2}d{{\boldsymbol{z}}}_{i}\wedge {\boldsymbol{K}}d{{\boldsymbol{z}}}_{i},{{\boldsymbol{z}}}_{i}={[{u}_{i},{\phi }_{i},{\omega }_{i},{\rho }_{i},{v}_{i}]}^{{\rm{T}}},$ index *i* represent *i* th equation and is from 1 to *N*.

### Proof.

Equation (3.3) can be re-written as a compact form

3.5 $${\boldsymbol{M}}\frac{d{{\boldsymbol{z}}}_{i}}{dt}+{\boldsymbol{K}}\mathop{\sum }\limits_{k=0}^{N-1}{({\boldsymbol{D}})}_{i,k}{{\bf{z}}}_{k}={\nabla }_{{\bf{z}}}S({{\boldsymbol{z}}}_{i}).$$

The variational equation associated with Eq. (3.5) is

3.6 $${\boldsymbol{M}}\frac{d}{dt}d{{\bf{z}}}_{i}+{\boldsymbol{K}}\mathop{\sum }\limits_{k=0}^{N-1}{({\boldsymbol{D}})}_{i,k}d{{\boldsymbol{z}}}_{k}={S}_{{\boldsymbol{zz}}}({{\boldsymbol{z}}}_{i})d{{\boldsymbol{z}}}_{i}.$$

Taking the wedge product with $d{{\boldsymbol{z}}}_{i}$ on both sides of Eq. (3.6) and noticing

3.7 $$d{{\boldsymbol{z}}}_{i}\wedge {S}_{{\boldsymbol{zz}}}({{\boldsymbol{z}}}_{i})d{{\boldsymbol{z}}}_{i}=0,$$

thus, we show the *N* semi-discrete multi-symplectic conservation laws.

Because ***D*** is anti-symmetry and ${{\boldsymbol{\zeta }}}_{i,k}={{\boldsymbol{\zeta }}}_{k,i}$, summing Eq. (3.6) over the spatial index yields

3.8 $$\frac{d}{dt}\mathop{\sum }\limits_{j=0}^{N-1}{{\boldsymbol{\chi }}}_{j}=0,$$

which implies conservation of the total symplecticity over time. Thus, it is natural to integrate with respect to time by using a symplectic integrator^13,29^.

Discretizing Eq. (3.3) with respect to the time domain by the symplectic Euler scheme yields

3.9 $${{\boldsymbol{M}}}_{+}{\delta }_{t}^{+}{{\boldsymbol{z}}}_{i}^{j}+{{\boldsymbol{M}}}_{-}{\delta }_{t}^{-}{{\boldsymbol{z}}}_{i}^{j}+{\boldsymbol{K}}\mathop{\sum }\limits_{k=0}^{N-1}{({\boldsymbol{D}})}_{i,k}{{\boldsymbol{z}}}_{k}^{j}={\nabla }_{{\boldsymbol{z}}}S({{\boldsymbol{z}}}_{i}^{j}),$$

where ${\delta }_{t}^{+}$ and ${\delta }_{t}^{-}$ are the forward and backward difference operators, respectively:

3.10 $${\delta }_{t}^{+}{{\boldsymbol{z}}}_{i}^{j}=\frac{{{\boldsymbol{z}}}_{i}^{j+1}-{{\boldsymbol{z}}}_{i}^{j}}{\tau },{\delta }_{t}^{-}{{\boldsymbol{z}}}_{i}^{j}=\frac{{{\boldsymbol{z}}}_{i}^{j}-{{\boldsymbol{z}}}_{i}^{j-1}}{\tau },$$

and ${{\boldsymbol{M}}}_{+}$ and ${{\boldsymbol{M}}}_{-}$ are the matrices splitting for the symplectic structure matrix ${\boldsymbol{M}}$,

3.11 $${\boldsymbol{M}}={{\boldsymbol{M}}}_{+}+{{\boldsymbol{M}}}_{-},\,{{\boldsymbol{M}}}_{+}^{{\rm{T}}}=-\,{{\boldsymbol{M}}}_{-}.$$

The matrix splitting of ${\boldsymbol{M}}$ is not unique^13,29^. Here, taking ${{\boldsymbol{M}}}_{+}$ as an upper triangle matrix

3.12 $${{\boldsymbol{M}}}_{+}=-\,{{\boldsymbol{M}}}_{-}^{{\rm{T}}}=[\begin{array}{cccc}0 & 1 & 0 & 0\\ 0 & 0 & 0 & 0\\ 0 & 0 & 0 & 0\\ 0 & 0 & 0 & 0\end{array}],$$

an efficient stable explicit scheme for the generalized KdV Eq. (1.2) is obtained as follows:

313 $$\begin{array}{c}\,\,(\frac{{u}_{i}^{j+1}-{u}_{i}^{j}}{\tau })-{\boldsymbol{D}}{({{\boldsymbol{\omega }}}_{k}^{j})}_{i}=0,\\ \,-(\frac{{\varphi }_{i}^{j}-{\varphi }_{i}^{j-1}}{\tau })-2\delta {\boldsymbol{D}}{({{\boldsymbol{v}}}_{k}^{j})}_{i}=\frac{2\alpha {({u}_{i}^{j})}^{\lambda +1}}{\lambda +1}+{\omega }_{i}^{j},\\ \,\,\,\,\,\,\,\,2\delta {({\boldsymbol{D}}{{\boldsymbol{u}}}_{k}^{j})}_{i}=2\delta {v}_{i}^{j},\\ \,\,\,\,\,\,\,\,\,\,\,{({\boldsymbol{D}}{{\boldsymbol{\Phi }}}_{k}^{j})}_{i}={u}_{i}^{j}.\end{array}$$

Eliminating the auxiliary variables and re-writing the equations, a compact form is obtained as follows:

314 $$\frac{1}{2}(\frac{{u}_{i}^{j+1}-{u}_{i}^{j-1}}{\tau })+\delta {({{\boldsymbol{D}}}^{3}{u}_{k}^{j})}_{i}+\alpha {({u}^{\lambda })}_{k}^{j}{({\boldsymbol{D}}{u}_{k}^{j})}_{i}=0,\,(i=1,\cdots ,N-1).$$

where *i* is spatial index and ***D***^3^ = ***D*** · ***D*** · ***D***.

### **Theorem 2**.

The discrete scheme (3.13) has *N* full-discrete multi-symplectic conservation laws

3.15 $$\frac{{{\boldsymbol{\chi }}}_{i}^{j}-{{\boldsymbol{\chi }}}_{i}^{j-1}}{\tau }+\mathop{\sum }\limits_{k=0}^{N-1}{({\boldsymbol{D}})}_{i,k}{{\boldsymbol{\zeta }}}_{k}^{j}=0,(i=1,2,\cdots ,N),$$

where ${{\boldsymbol{\chi }}}_{i}^{j}=\frac{1}{2}d{{\boldsymbol{z}}}_{i}^{j}\wedge {{\boldsymbol{M}}}_{+}d{{\boldsymbol{z}}}_{i}^{j+1},{{\boldsymbol{\zeta }}}_{k}^{j}=\frac{1}{2}d{{\boldsymbol{z}}}_{i}^{j}\wedge {\boldsymbol{K}}d{{\boldsymbol{z}}}_{k}^{j},$ index *i* representation *i* th equation.

### **Proof**

From theorem 1 and Eqs (3.6) and (3.13) can be re-written as a compact form

3.16 $${{\boldsymbol{M}}}_{+}\frac{{{\boldsymbol{z}}}_{i}^{j+1}-{{\boldsymbol{z}}}_{i}^{j}}{\tau }+{{\boldsymbol{M}}}_{-}\frac{{{\boldsymbol{z}}}_{i}^{j}-{{\boldsymbol{z}}}_{i}^{j-1}}{\tau }+{\boldsymbol{K}}\mathop{\sum }\limits_{k=0}^{N-1}{({\boldsymbol{D}})}_{i,k}{{\boldsymbol{z}}}_{k}^{j}={\nabla }_{{\bf{z}}}S({{\boldsymbol{z}}}_{i}^{j}).$$

The variational equation associated with Eq. (3.16) is

3.17 $${{\boldsymbol{M}}}_{+}\frac{d{{\boldsymbol{z}}}_{i}^{j+1}-d{{\boldsymbol{z}}}_{i}^{j}}{\tau }+{{\boldsymbol{M}}}_{-}\frac{d{{\boldsymbol{z}}}_{i}^{j}-d{{\boldsymbol{z}}}_{i}^{j-1}}{\tau }+{\boldsymbol{K}}\mathop{\sum }\limits_{k=0}^{N-1}{({\boldsymbol{D}})}_{i,k}d{{\boldsymbol{z}}}_{k}^{j}={S}_{{\boldsymbol{zz}}}({{\boldsymbol{z}}}_{i}^{j})d{{\boldsymbol{z}}}_{i}^{j}.$$

Taking the wedge product with $d{{\boldsymbol{z}}}_{i}^{j}$ on both sides of Eq. (3.17) and noticing

3.18 $$d{{\boldsymbol{z}}}_{i}^{j}\wedge {S}_{{\boldsymbol{zz}}}({{\boldsymbol{z}}}_{i}^{j})d{{\boldsymbol{z}}}_{i}^{j}=0.$$

then, the *N* full-discrete multi-symplectic conservation laws are verified^13,29^.

## Numerical Experiment

In this section, we conduct a typical numerical experiment for the scheme (3.14) to verify the theoretical conclusions, including the accuracy, the ability to preserve the local energy conservation law and the local momentum conservation law of the generalized KdV Eq. (1.2) for long-time integration.

Applying the Riccati-Bernoulli sub-ODE method^30^ to the generalized KdV Eq. (1.2), has an exact solution as follows:

3.19 $$u={(\frac{A\delta ({\lambda }^{2}+3\lambda +2)}{2\alpha \lambda }s{{\rm{ech}}}^{2}(\frac{\sqrt{A}}{2}\xi ))}^{\frac{1}{\lambda }},\xi =x-\frac{A\delta }{{\lambda }^{2}}t,$$

where *A* is an arbitrary constant.

Inserting the parameters $\lambda =\sqrt{2},\alpha =1,\delta =1$ and $A=2$ into Eq. (1.2), we obtain

3.20 $${u}_{t}+{u}^{\sqrt{2}}{u}_{x}+{u}_{xxx}=0,$$

which has the exact traveling wave solution (set $\lambda =\sqrt{2},\alpha =1,\delta =1$ and *A* = 2 in Eq. (3.19))

3.21 $$u(x,t)={(\frac{(4+3\sqrt{2})}{2}{\mathrm{Sech}}^{2}(\frac{\sqrt{2}}{2}(x-t)))}^{\frac{1}{\sqrt{2}}}.$$

The space interval is $[{x}_{L},{x}_{R}]=[-\,20,40]$ with the periodic boundary condition

3.22 $$u(x-20,t)=u(x+40,t),$$

and the initial condition

3.23 $$u(x,0)={(\frac{(4+3\sqrt{2})}{2}{\mathrm{Sech}}^{2}(\frac{\sqrt{2}}{2}x))}^{\frac{1}{\sqrt{2}}}.$$

We fix the space step $h=0.2$ and the time step $\tau =1\times {10}^{-4}$ for the scheme (3.14). Based on the multi-symplectic theory of Bridges^24–27^, the global energy *E*(*t*) and the global momentum *I*(*t*) of the generalized KdV Eq. (3.20) with the periodic boundary condition (3.22) and the initial condition (3.23) are written as

3.24 $$E(t)={\int }_{-20}^{40}(\frac{2{u}^{\sqrt{2}+2}}{4+3\sqrt{2}}-{u}_{x}^{2})dx,I(t)=-{\int }_{-20}^{40}{u}^{2}dx.$$

Accordingly, *E*^*j*^ and *I*^*j*^, which denote the discrete global energy and the global momentum on the *j*th time level, are written as

3.25 $$\begin{array}{c}{E}^{j}=\frac{1}{5}\mathop{\sum }\limits_{i=1}^{N}(\frac{2}{4+3\sqrt{2}}{(\frac{{u}_{i}^{j}+{u}_{i-1}^{j}}{2})}^{\sqrt{2}+2}-{(\frac{{({\boldsymbol{D}}{{\boldsymbol{u}}}_{k}^{j})}_{i}+{({\boldsymbol{D}}{{\boldsymbol{u}}}_{k}^{j})}_{i-1}}{2})}^{2}),\\ {I}^{j}=-\,\frac{1}{5}{\mathop{\sum }\limits_{i=1}^{N}(\frac{{u}_{i}^{j}+{u}_{i-1}^{j}}{2})}^{2}.\end{array}$$

The errors of the discrete global energy conservation law Error *E* and the global momentum conservation law Error *I* on the *j*th time level are defined as follows:

3.26 $${\rm{Error}}\,E={E}^{j}-{E}^{0},\,{\rm{Error}}\,I={I}^{j}-{I}^{0},$$

where ${E}^{0}$ and ${I}^{0}$ are the initial value of the discrete global energy and the global momentum.

Now we define the error as follows:

3.27 $${\rm{Error}}=\mathop{{\rm{\max }}}\limits_{0\le i\le N-1}{\Vert u({x}_{i},j\tau )-{u}_{i}^{j}\Vert }_{\infty }.$$

**Figure 1:**
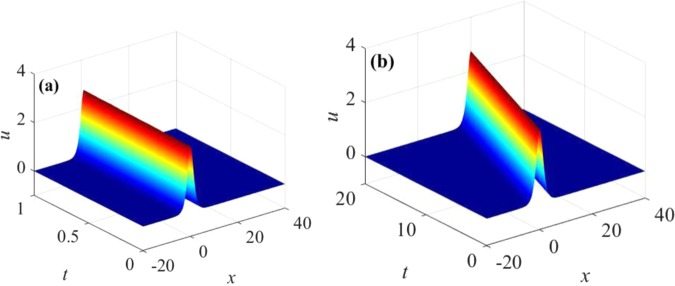
Three-dimensional waveform of numerical solution: (**a**) the numerical solution with time $0\le t\le 1$, (**b**) the numerical solution with time $0\le t\le 20$.

**Figure 2:**
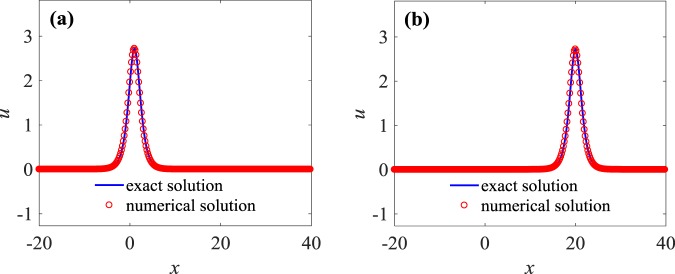
Two-dimensional waveform of numerical solution and exact solution: (**a**) The exact solution and the numerical solution with time *t* = 1, (**b**) The exact solutions and the numerical solutions with time *t* = 20.

**Figure 3:**
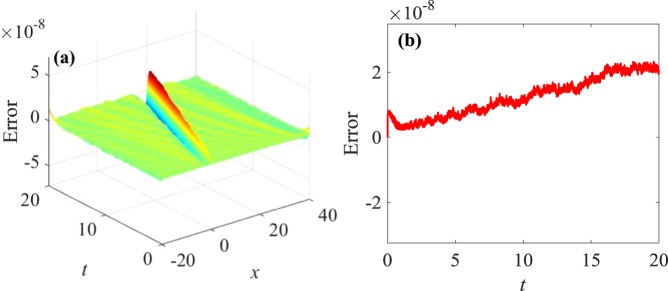
Errors with time $0\le t\le 20$: (**a**) three-dimensional errors with time, (**b**) two-dimensional errors with time.

**Figure 4:**
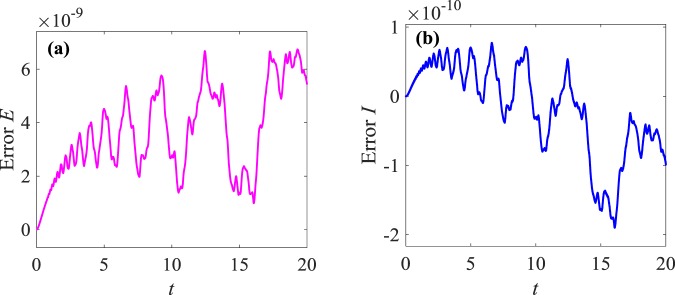
Errors of the discrete global conservation laws with time $0\le t\le 20$: (**a**) two-dimensional errors of the discrete global energy conservation law (**b**) two-dimensional errors of discrete global momentum conservation law.

Applying the scheme (3.14) to simulate the generalized KdV Eq. (3.20) with the periodic boundary condition (3.22) and the initial conditions (3.23) up to *t* = 20, three-dimensional waveform figure of numerical solutions (Fig. 1a (*t* = 1) and Fig. 1b (*t* = 20)), two-dimensional waveform figure of numerical solutions (Fig. 2a (*t* = 1) and Fig. 2b (*t* = 20)), three-dimensional error figure (Fig. 3a), two-dimensional error figure (Fig. 3b), the global energy error figure (Fig. 4a) and the global momentum error figure (Fig. 4b) are obtained as follows:

Figure 1 shows that waveform of numerical solutions does not change with time by applying the multi-symplectic Fourier pseudo-spectral method to Eq. (3.20). It indicates that the basic geometric properties of Eq. (3.20) can be well maintained by the numerical soliton solutions. From Fig. 2a,b, *u* decreases gradually and tends to zero with $x\to \infty$, which is consistent with the exact solution (3.21), and the numerical solutions nearly overlap the exact solution, which implies high accuracy of the multi-symplectic Fourier pseudo-spectral scheme. From the Fig. 3a,b, it is shown that the errors between the exact solution and the numerical solution are up to 10^−9^, and the errors are mainly from the boundary conditions. From Fig. 4a,b, the multi-symplectic Fourier pseudo-spectral method to the generalized KdV equation can well maintain two important geometric properties of the system, which are the global energy conservation law and the global momentum conservation law. Obviously, when we take 10^−5^ time steps, the multi-symplectic Fourier pseudo-spectral method to the generalized KdV equation can preserve the discrete global energy conservation law and global momentum conservation law quite well, which implies long-time numerical stability of the multi-symplectic pseudo-spectral method.

## Conclusions

The generalized KdV-type equation, which can degenerate to the mKdV equation and the generalized KdV equation, is given. The variational principle is successfully used to establish a multi-symplectic structure for the KdV-type equation. Based on the variational principle, we also obtain the multi-symplectic structure, local energy conservation law and local momentum conservation law of the generalized KdV-type equation, which are identical to the results by using the HBUCM^6,13^. An explicit multi-symplectic scheme for the generalized KdV equation based on the Fourier pseudo-spectral method and the symplectic Euler scheme are constructed. Through a numerical examination, the explicit multi-symplectic Fourier pseudo-spectral scheme for the generalized KdV equation not only preserve the discrete global energy conservation law and the global momentum conservation law with high accuracy, but show long-time numerical stability as well.

The performance of variational principle is found to be simple and efficient. Moreover, similar to the process of the generalized KdV-type equation, multi-symplectic structures of some NLPDEs can be obtained.
